# Utilization of Point-of-Care Ultrasound in Routine Patient Care Among Internal Medicine Resident Physicians in the United States

**DOI:** 10.1016/j.ajmo.2026.100133

**Published:** 2026-04-30

**Authors:** Ugochukwu Ebubechukwu, Onyinye Ugoala, Chinenye Okafor, Festus Ibe, Duaa Mohamed, Anderson Anuforo, Sahil Chaddha, Ayesha Samad, Oboseh John Ogedegbe, Ahmad Abdelkhalek, Samy I. McFarlane, Pooja Belligund

**Affiliations:** a SUNY Downstate Health Sciences University, Brooklyn, NY; b Texas Tech Health Sciences University, Amarillo, TX; c Medical University of South Carolina Regional Network, Florence, SC; d Jefferson Einstein Hospital, Philadelphia, PA; e SUNY Upstate Medical University, Syracuse, NY; f Ocean University Medical Center, Brick, NJ; g Trinity Health, Ann Arbor, MI; h Northwestern Medicine McHenry Hospital/Rosalind Franklin University, McHenry, IL; i Veterans Affairs New York Harbor Healthcare System Brooklyn Campus, NY

**Keywords:** Internal medicine, Medical education, POCUS curriculum, Point-of-care ultrasound (POCUS), Residents, United States

## Abstract

**Background:**

The integration of point-of-care ultrasound (POCUS) into internal medicine (IM) residency training and curriculum remains an area for further development and research.

**Objective:**

To assess the confidence and barriers to the utilization of POCUS among IM residents in the United States.

**Methods:**

This cross-sectional, multi-institutional study involved residents from various IM programs across the United States, who were invited to participate in a voluntary online survey on POCUS confidence and utilization between October 30, 2024, and December 3, 2024.

**Results:**

Out of 273 respondents (16.2%), 96.3% were IM residents, and 50.2% were female. 34.4%, 41.0%, and 24.5% were postgraduate year (PGY)-1, PGY-2, and PGY-3, respectively. Residents felt least comfortable with and wanted to learn focused cardiac (46.9%) and extended focused assessment with sonography in trauma (45.1%) scans. The top barriers to POCUS utilization were lack of time (55.7%), lack of confidence in interpreting images (42.9%), and limited access to machines (36.3%). Desired interventions included designated elective time for POCUS training (65.6%), ultrasound machines on every floor (55.3%), and formal integration of POCUS training into residency (53.5%). Self-reported confidence also correlated with training level.

**Conclusions:**

This is the first national survey to highlight critical gaps in POCUS utilization among IM residents. This study emphasizes the need for targeted POCUS interventions from program leaders, professional societies, and accrediting bodies for IM residents.

## Highlights

• POCUS enhances diagnostic accuracy, assists in clinical management, and may reduce healthcare costs by facilitating faster diagnoses and decreasing the length of hospital stays.
• Despite increasing awareness of POCUS across most Internal Medicine (IM) residency programs, residents across all program types face significant challenges that hinder its effective use.
• Collective efforts from IM program leaders and professional medical societies can improve the utilization of POCUS in IM residency programs nationwide.

## Introduction

Point-of-care ultrasound (POCUS) has emerged as a valuable diagnostic tool in various medical settings, particularly critical care and emergency medicine (EM), as it allows physicians to integrate findings for clinical decision-making immediately.^1^ Advancements in technology, portability, and affordability of ultrasound machines have expanded the utility and scope of POCUS, making it increasingly accessible to various medical specialties, including internal medicine (IM).

Studies have demonstrated that POCUS can improve diagnostic accuracy, guide clinical management, and potentially reduce healthcare costs by facilitating quicker diagnoses and shorter hospital stays.^2^ However, the integration of POCUS into IM practice and training has been relatively slow, with significant barriers to its widespread adoption. Despite the growing recognition of its benefits, structured POCUS curricula have only recently begun to be incorporated into IM residency programs. While many IM program directors acknowledge the importance of formal POCUS training and many learners express a strong desire for it, challenges persist, including the lack of standardized guidelines and the limited experience of most programs in effectively teaching these skills.^3^^,^^4^

The need for a formal, integrated curriculum and adequate training for the safe and effective use of POCUS has been recognized.^5^ For example, the Accreditation Council for Graduate Medical Education has established POCUS competency requirements for EM residents, and the American College of Emergency Physicians has released guidelines and recommendations for POCUS education.^6^ Similarly, the American Society of Anesthesiologists has published recommendations regarding the scope of practice and training in POCUS for anesthesiologists and trainees.^7^ In the context of IM, however, the integration of POCUS into resident training and routine clinical practice remains an area for further development and research. This study aimed to explore the utilization of POCUS among IM resident physicians in routine patient care and to examine the challenges associated with its implementation.

## Methods

This study was reported in accordance with the Standards for Quality Improvement Reporting Excellence in Education guidelines.^8^

### Study Design and Participants

A cross-sectional, multi-institutional online survey was administered to IM residents between October 30, 2024, and December 3, 2024. IM residents from 27 residency programs (including IM-EM residents at one program only) in multiple states within the United States were invited to participate in the survey. The SUNY Downstate Health University institutional review board approved this study as exempt.

### Survey Development

The modified survey, which had been validated and utilized in other research studies,^9^ was reviewed by ultrasound faculty members. To further ensure content validity, following an external review, the original survey was further modified to fit the targeted audience, ie, IM residents. In addition, based on feedback from a few residents during a test run, some irrelevant or confusing questions were removed to further refine the original survey. The survey consisted of 23 items, including multiple-choice questions with single or multiple answers and a 5-point Likert scale (strongly disagree, disagree, neutral, agree, strongly agree) to assess respondents’ knowledge of the different practices and barriers to effective utilization of POCUS in their respective institutions, and their confidence using POCUS. The survey was distributed via a Google questionnaire. All responses were kept anonymous. The complete survey is available in the Supplementary Data.

### Data Collection

The survey was distributed electronically via academic email and other closed, engaging platforms such as WhatsApp, LinkedIn, and X (formerly Twitter) to verified IM chief residents and residents. The IM chief residents and residents were verified through the programs’ official websites and social media pages. In addition, some program directors were contacted via their academic email addresses. The contacted residents/chief residents/program leaders were encouraged to share the survey with the residents in their different residency programs. The survey was voluntary, and no monetary incentives were offered to respondents. The invitation to participate in the survey included a cover letter describing the study’s purpose, a link to the survey, and the contact information for the principal investigator and the IRB for questions or concerns.

The study population for each institution was estimated using data from the Association of American Medical Colleges Residency Explorer tool. Respondents included IM residents from the following US states: Connecticut, Florida, Georgia, Illinois, Louisiana, Maryland, Massachusetts, Michigan, Missouri, New Jersey, New York, Ohio, Pennsylvania, South Carolina, Tennessee, Texas, Washington, DC, and West Virginia.

Data on demographic characteristics included categories of age and self-reported sex. Information on respondents’ program characteristics included program type (community, university, or university-affiliated), as reported on the Association of American Medical Colleges Residency Explorer, postgraduate year (PGY), specialty of training, and respondents’ knowledge of having ultrasound machines on the medical floor, as well as whether their programs had an ultrasound medical director. Respondents’ prior POCUS training and knowledge of the POCUS curriculum within their programs were also collected. Specifically, information was gathered on whether a POCUS curriculum existed to educate and train residents, a credentialing pathway for both untrained and experienced users, a CME (CME) pathway to maintain credentialing, and a quality assurance management system for the curriculum. Confidence levels in utilizing POCUS were evaluated for adjusting basic settings, selecting appropriate probes, locating and achieving vascular access, conducting a focused assessment with sonography in trauma (FAST) exam or extended FAST (e-FAST), diagnosing pericardial effusion, identifying tamponade, detecting pneumothorax, diagnosing hemoperitoneum or ascites, assessing volume responsiveness, evaluating left ventricular (LV) function, assessing right ventricular function and volume overload, diagnosing shock, and for overall interpretation of POCUS results. Confidence in obtaining the parasternal long axis view, parasternal short axis views, apical four-chamber view, apical five-chamber view, inferior vena cava (IVC) view, and subcostal view were also collected. Information on the types of scans (e-FAST, focused cardiac, IVC volume assessment, soft tissue, or procedural) was provided for those the respondents performed, those they were less comfortable with, and those they would like to learn more about. Barriers to POCUS utilization were assessed independently, including a lack of time, access to a machine, ultrasound medical director, ultrasound curriculum, support, confidence in obtaining images, confidence in interpreting images, quality assurance, and faculty availability.

### Statistical Analysis

PGY levels above three (ie, above PGY-3) were defined as “enhanced-skill residents” and were excluded from the overall analysis. Demographics, respondents’ program characteristics, and their years of POCUS experience were summarized and presented as percentages. Responses on the Likert scale were reported as percentages. “Strongly agree” and “agree” were then combined, as were “strongly disagree” and “disagree,” for further comparative analysis. Data analysis was carried out using Microsoft Excel and SAS software, version 9.4 (SAS Institute Inc., Cary, NC, USA). Only descriptive analyses were performed.

## Results

### Respondents Characteristics

A total of 276 residents completed the survey. After excluding responses from enhanced-skill residents, 273 out of the expected 1684 resident physicians (response rate of 16.2%) from 27 residency programs were included in the study. 34.4%, 41.0%, and 24.5% were PGY-1, PGY-2, and PGY-3, respectively. 96.3% were IM residents and 3.7% were IM-EM residents, and 50.2% were female. 45.4% of respondents were undergoing training in a university program, 26.4% were in a community program, and 24.5% were in a university-affiliated program (Table 1).

**Table 1:** Summary of Respondents’ Characteristics.

Variable	Number (Percentage)
Age	
18-24 y	5 (1.8)
25-34 y	227 (83.2)
35-44 y	40 (14.7)
45 y and above	1 (0.4)
Sex	
Female	137 (50.2)
Male	136 (49.8)
Specialty	
Internal medicine	263 (96.3)
Internal medicine-emergency medicine	10 (3.7)
Level of training	
PGY-1	94 (34.4)
PGY-2	112 (41.0)
PGY-3	67 (24.5)
Program type	
Community program	72 (26.4)
University program	124 (45.4)
University-affiliated program	67 (24.5)
Years of POCUS experience	
0-1 y	191 (70.0)
2-3 y	71 (26.0)
4-5 y	5 (1.8)
5 or more y	4 (1.5)
USS medical director	
Yes	132 (48.4)
No	97 (35.5)
Not sure	44 (16.1)
USS on every medical floor	
Yes	92 (33.7)
No	154 (56.4)
Not sure	27 (9.9)

Data limited to PGY 1-3.

PGY = postgraduate year; POCUS = point-of-care ultrasound; USS = ultrasound.

### Utilization of POCUS

**Figure 1:**
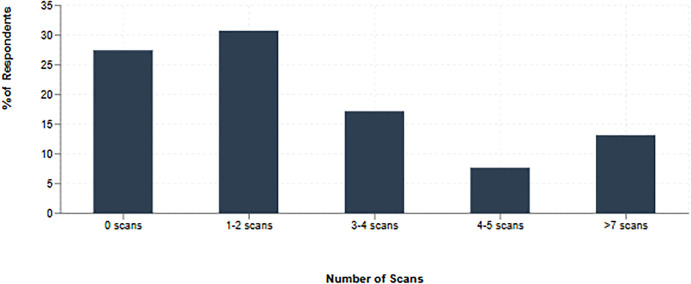
Number of point-of-care ultrasound (POCUS) scans in the past month.

**Figure 2:**
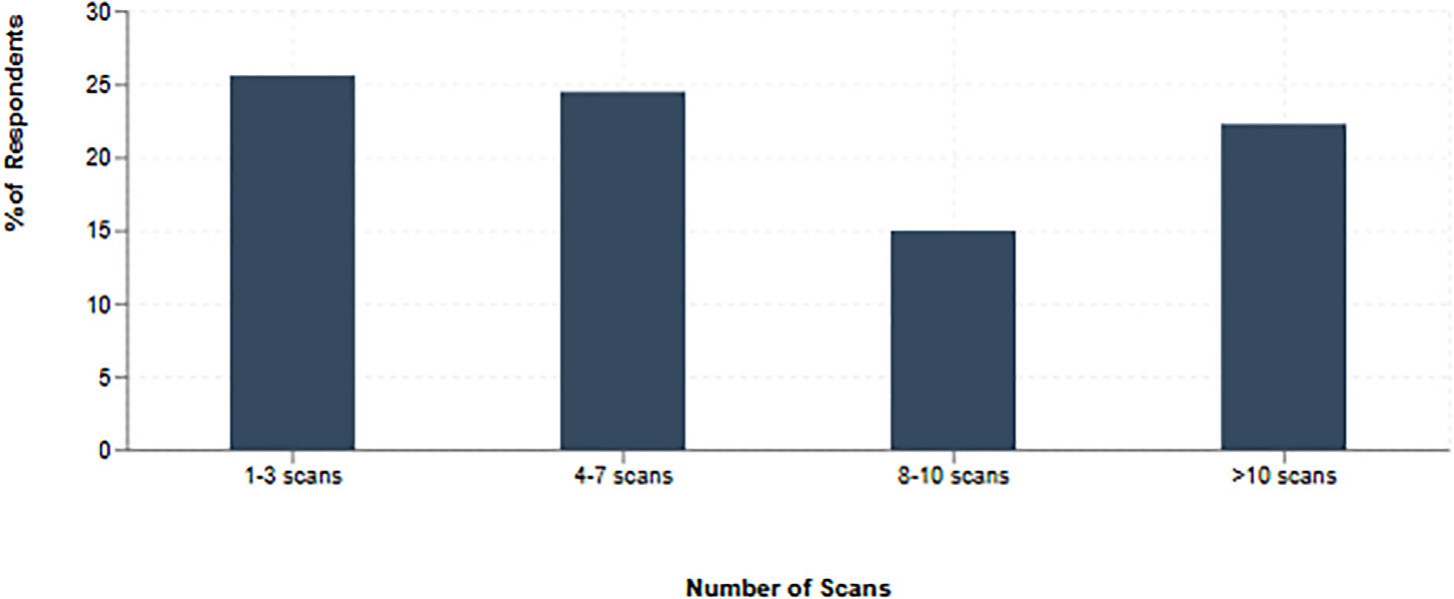
Number of point-of-care ultrasound (POCUS) scans in the past 3 months.

**Figure 3:**
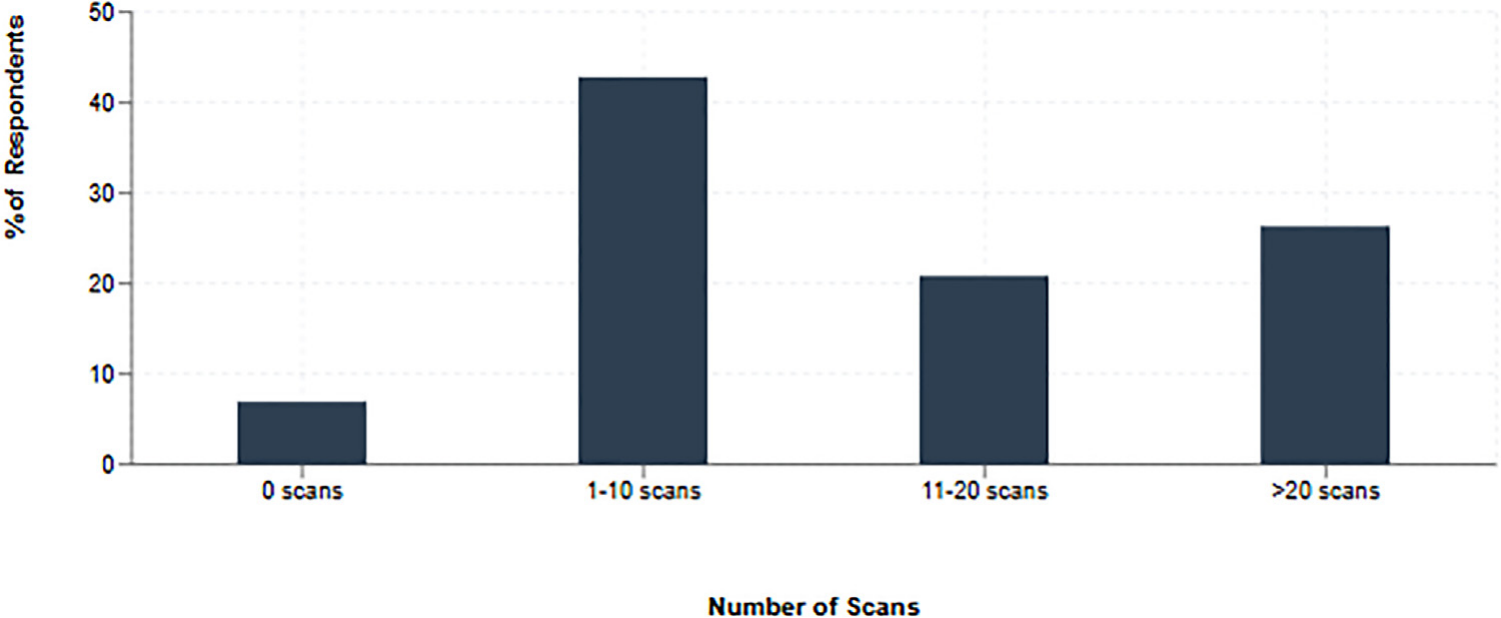
Number of point-of-care ultrasound (POCUS) scans in the past 12 months.

**Figure 4:**
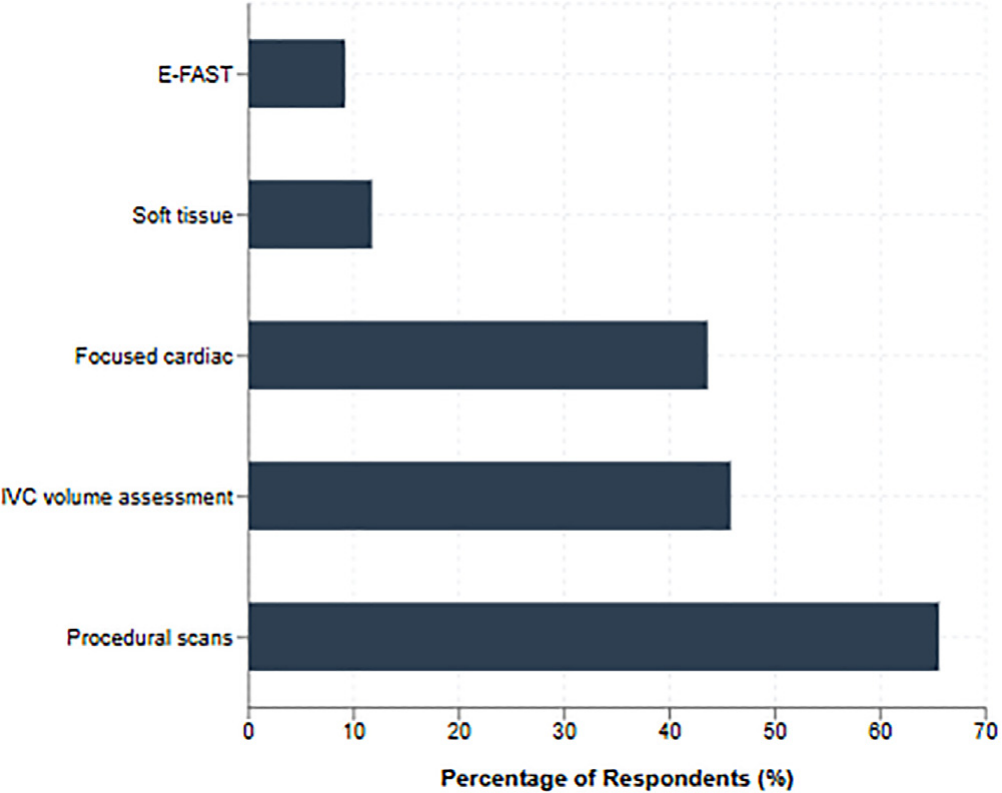
Most frequently performed point-of-care ultrasound (POCUS) scans.

**Figure 5:**
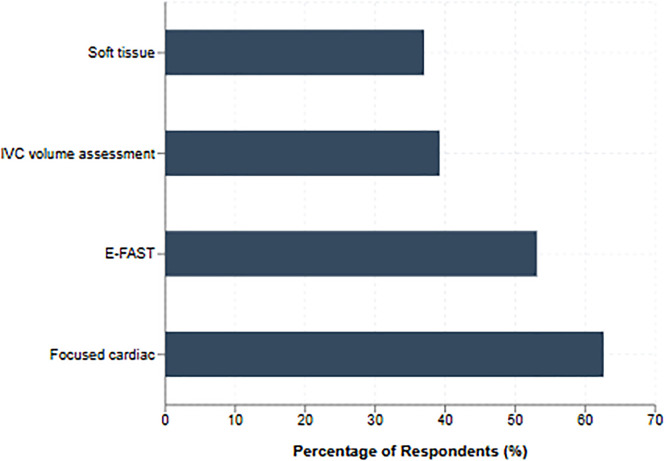
Point-of-care ultrasound (POCUS) scans residents wish to learn.

Approximately 58.2% of residents reported scanning only two or fewer patients in the past month. In the past 3 months, 50.2% scanned 1-7 patients; during the past year, 42.9% scanned 1-10 patients, and 26.4% scanned more than 20 patients (Figure 1, Figure 2, Figure 3). The most frequently performed scans were procedural (65.6%) and IVC volume assessments (45.8%). However, residents reported feeling least comfortable with focused cardiac (46.9%) and e-FAST (45.1%) scans, which also ranked as the top scans residents wished to learn (62.6% and 53.1%, respectively) (Figure 4, Figure 5).

### Barriers to POCUS Utilization and Suggested Interventions to Improve Utilization

**Figure 6:**
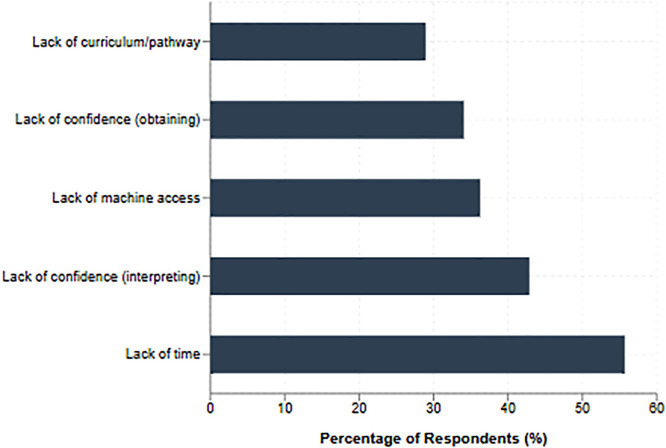
Barriers to the utilization of point-of-care ultrasound (POCUS) internal medicine residency programs.

**Figure 7:**
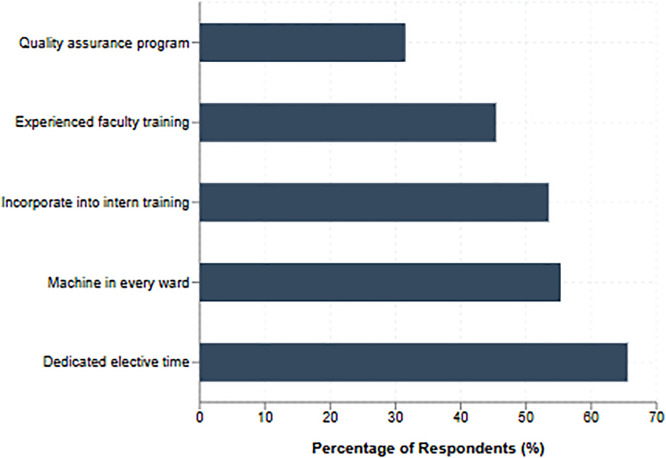
Desired interventions to improve point-of-care ultrasound (POCUS) utilization in internal medicine residency programs.

Common barriers to POCUS utilization included a lack of time (55.7%), a lack of confidence in interpreting images (42.9%), limited access to machines (36.3%), and a lack of confidence in obtaining images (34.1%) (Figure 6). Desired interventions included designated elective time for POCUS training (65.6%), ensuring an ultrasound machine on every floor (55.3%), formal integration of POCUS training into the residency program (53.5%), and experienced faculty for hands-on teaching (45.4%) (Figure 7).

### Residents’ Confidence in the Utilization of POCUS

Full details of respondents’ confidence in utilizing POCUS are presented in Table 2. Specifically, 30.8% and 35.9% of respondents strongly agree and agree, respectively, that they were confident in adjusting basic ultrasound knobs, with a similar pattern observed in their confidence in selecting appropriate ultrasound probes (37.7% agree and 33.7% strongly agree) and in finding and obtaining vascular access (35.2% agree and 27.8% strongly agree). However, a majority of the residents (53.8%) responded disagree or strongly disagree to confidence in performing a FAST/e-FAST exam. A similar trend continued, with confidence dropping sharply as the majority of residents responded with a “disagree” or “strongly disagree” assessment of RV function (43.6%), diagnosing tamponade (42.5%), pneumothorax (42.2%), or shock (48.0%). Only 5.5% and 19.1% of respondents strongly agree and agree, respectively, that they were confident in their overall POCUS interpretation.

**Table 2:** Summary of Likert Scale Responses.

Variable	Strongly Disagree	Disagree	Neutral	Agree	Strongly Agree
Confidence in;					
Adjusting basic knobs	16 (5.9)*	22 (8.1)	53 (19.4)	98 (35.9)	84 (30.8)
Selecting the correct probe	14 (5.1)	20 (7.3)	44 (16.1)	103 (37.7)	92 (33.7)
Finding and obtaining vascular access	12 (4.4)	19 (7.0)	70 (25.6)	96 (35.2)	76 (27.8)
Performing a FAST exam or e-FAST	53 (19.4)	94 (34.4)	75 (27.5)	33 (12.1)	18 (6.6)
Diagnosing pericardial effusion	27 (9.9)	61 (22.3)	79 (28.9)	74 (27.1)	32 (11.7)
Diagnosing tamponade	30 (11.0)	86 (31.5)	76 (27.8)	58 (21.3)	23 (8.4)
Diagnosing a pneumothorax	31 (11.4)	84 (30.8)	83 (30.4)	49 (18.0)	26 (9.5)
Diagnosing hemoperitoneum or ascites	34 (12.5)	59 (21.6)	74 (27.1)	69 (25.3)	37 (13.6)
Assessing left ventricular function	32 (11.7)	58 (21.3)	88 (32.2)	66 (24.2)	29 (10.6)
Assessing right ventricular function and volume overload	32 (11.7)	87 (31.9)	77 (28.2)	62 (22.7)	15 (5.5)
Assessing volume responsiveness	30 (11.0)	65 (23.8)	82 (30.0)	74 (27.1)	22 (8.1)
Diagnosing shock	39 (14.3)	92 (33.7)	76 (27.8)	52 (19.1)	14 (5.1)
Obtaining the parasternal long-axis view	29 (10.6)	48 (17.6)	75 (27.5)	94 (34.4)	27 (9.9)
Obtaining parasternal short-axis view (mitral valve level)	29 (10.6)	58 (21.3)	80 (29.3)	83 (30.4)	23 (8.4)
Obtaining parasternal short-axis view (aortic valve level)	30 (11.0)	66 (24.2)	80 (29.3)	73 (26.7)	24 (8.8)
Obtaining the apical four-chamber view	28 (10.3)	55 (20.2)	89 (32.6)	81 (29.7)	20 (7.3)
Obtaining an apical five-chamber view	34 (12.5)	61 (22.3)	100 (36.6)	62 (22.7)	16 (5.9)
Obtaining the subcostal view	31 (11.4)	50 (18.3)	92 (33.7)	80 (29.3)	20 (7.3)
Obtaining the IVC view	28 (10.3)	50 (18.3)	79 (28.9)	92 (33.7)	24 (8.8)
Overall POCUS interpretation	34 (12.5)	70 (25.6)	102 (37.4)	52 (19.1)	15 (5.5)

*Values are represented as number (percentage).

e-FAST = extended focused assessment with sonography in trauma; FAST = focused assessment with sonography in trauma; IVC = inferior vena cava; *N* = number; POCUS = point-of-care ultrasound.

### Utilization and Confidence Trends by PGY Level

When examined by PGY level (PGY-1 vs PGY-2 vs PGY-3), the utilization patterns for types of scans performed were noted to increase steadily, notably for focused cardiac scans (40.4% vs 43.8% vs 47.8%), IVC assessment (38.3% vs 49.1% vs 50.8%), and procedural scans (57.5% vs 68.8% vs 71.6%). Minimal use of e-FAST and soft-tissue scans was noted at all levels (Table 3).

**Table 3:** Utilization and Confidence in POCUS By PGY Level.

Variable	PGY-1 (*n* = 94)	PGY-2 (*n* = 112)	PGY-3 (*n* = 67)
Confidence in assessing left ventricular function			
Strongly disagree/disagree	41 (43.6)*	31 (27.7)	18 (26.9)
Neutral	28 (29.8)	44 (39.3)	16 (23.9)
Agree/strongly agree	25 (26.6)	37 (33.0)	33 (49.3)
Confidence in assessing volume responsiveness			
Strongly disagree/disagree	43 (45.7)	36 (32.1)	16 (23.9)
Neutral	27 (28.7)	36 (32.1)	19 (28.4)
Agree/strongly agree	24 (25.5)	40 (35.7)	32 (47.8)
Confidence in diagnosing shock			
Strongly disagree/disagree	53 (56.4)	51 (45.5)	27 (40.3)
Neutral	23 (24.5)	34 (30.4)	19 (28.4)
Agree/strongly agree	18 (19.2)	27 (24.1)	21 (31.3)
Confidence in the overall interpretation of POCUS			
Strongly disagree/disagree	49 (52.1)	39 (34.8)	16 (23.9)
Neutral	27 (28.7)	49 (43.8)	26 (38.8)
Agree/strongly agree	18 (19.2)	24 (21.4)	25 (37.3)
Types of scans performed			
e-FAST	8 (8.5)	10 (8.9)	7 (10.5)
Focused cardiac	38 (40.4)	49 (43.8)	32 (47.8)
IVC volume assessment	36 (38.3)	55 (49.1)	34 (50.8)
Soft tissue	8 (8.5)	14 (12.5)	10 (14.9)
Procedural	54 (57.5)	77 (68.8)	48 (71.6)
Types of scans less comfortable with			
e-FAST	39 (41.5)	50 (44.6)	34 (50.8)
Focused cardiac	51 (54.3)	55 (49.1)	22 (32.8)
IVC volume assessment	16 (17.0)	28 (25.0)	9 (13.4)
Soft tissue	29 (30.9)	40 (35.7)	26 (38.8)
Procedural	30 (31.9)	20 (17.9)	9 (13.4)
Types of scans would like to learn			
e-FAST	54 (57.5)	55 (49.1)	36 (53.7)
Focused cardiac	63 (67.0)	70 (62.5)	38 (56.7)
IVC volume assessment	43 (45.7)	45 (40.2)	19 (28.4)
Soft tissue	40 (42.6)	39 (34.8)	22 (32.8)
Procedural	45 (47.9)	34 (30.4)	19 (28.4)

*Values are represented as number (percentage).

e-FAST, extended focused assessment with sonography in trauma; IVC, inferior vena cava; *N*, number; PGY, postgraduate year; POCUS, point-of-care ultrasound.

**Figure 8:**
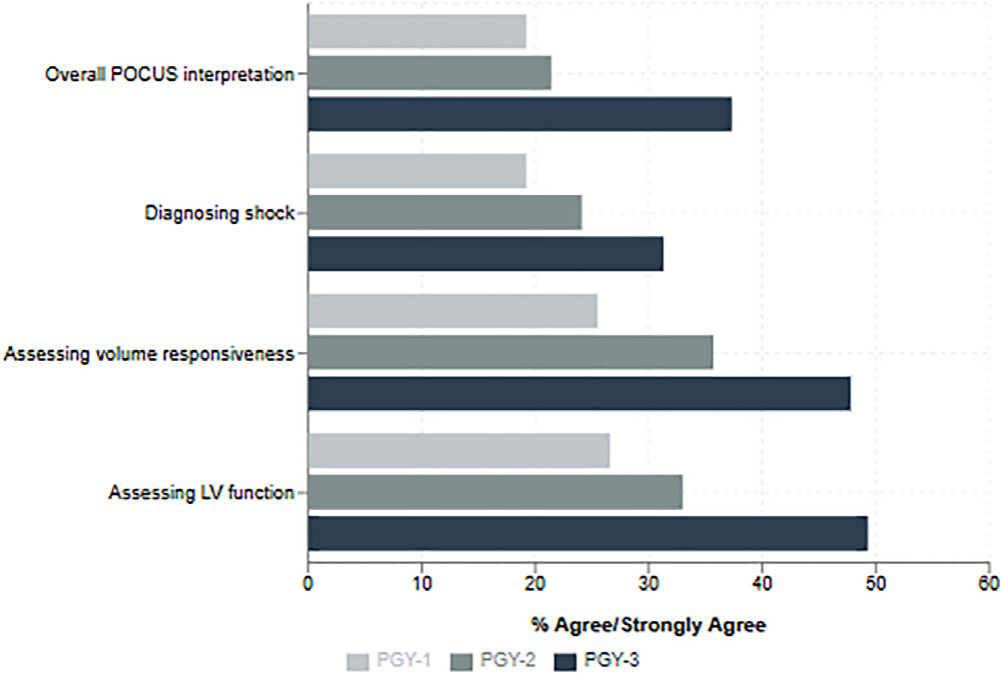
Self-reported confidence trends in POCUS skills by postgraduate year (PGY) level in internal medicine residency programs.

Similarly, confidence increased steadily with each training year, especially in assessing LV function (26.6% vs 33.0% vs 49.3%) and volume responsiveness. A gradual increase in confidence, though generally low, was also observed for shock assessment (19.2% vs 24.1% vs 31.3%) and overall POCUS interpretation (19.2% vs 21.4% vs 37.3%) (Figure 8).

## Discussion

To the best of our knowledge, this is the first national survey of POCUS utilization among IM residents in the United States. We demonstrated that despite the increasing awareness of POCUS across most residency programs, IM residents continue to face significant barriers to the utilization of POCUS. Our study highlights significant variability in POCUS utilization and confidence among IM residents.

POCUS, much like physical examination, requires teaching the indications, skills for image acquisition and interpretation, and clinical integration. The survey sought to assess residents’ familiarity with POCUS exams, program faculty, and accreditation pathways. Our findings revealed that POCUS use was generally limited. The majority (58%) of residents had scanned fewer than three patients in the prior month, and only a quarter had scanned more than 20 patients in the past year. Over 60% of the scans were procedural. The most common use of POCUS in clinical assessment was IVC volume assessment (45%). This may reflect either comfort level with the procedure or the need for an objective data point for residents to determine a patient’s fluid status. The increased IVC utilization may be tied to Society of Critical Care Medicine hemodynamic/vol assessment Bundle requirements, which are tied to CMS SEP-1 requirements, encouraging residents to obtain IVCs to guide fluid administration.^10^^,^^11^ There was also an interest in learning e-FAST and Focused Cardiac examinations (Lung ultrasound use was surveyed as the e-FAST tool, rather than the BLUE protocol or Lung ultrasound). The use of lung ultrasound for cardiogenic pulmonary edema was not specifically surveyed. This may have resulted in underreporting of Lung ultrasound use in pulmonary edema, and pneumonia use was not specified in our survey; thus, Lung ultrasound may be significantly underreported.

Most respondents cited lack of time as the most common barrier. This may reflect higher patient volume in some medical centers and competing academic demands. Generally, residents were also more likely to cite a lack of access to machines, ultrasound leadership, and structured curriculum. These findings are consistent with prior work, which identifies time constraints, lack of supervision, limited availability of ultrasound machines, and the absence of formal pathways as the most pervasive barriers to POCUS integration.^12^, ^13^, ^14^, ^15^

As expected, training level correlated with POCUS confidence. PGY-3 residents were more confident than PGY-1s across key competencies, including LV function assessment, volume responsiveness, shock evaluation, and overall interpretation of POCUS. Although overall confidence increased progressively with each training year, it remained < 40%, highlighting a persistent limitation in diagnostic interpretation. This finding aligns with studies demonstrating that POCUS skill acquisition is a progressive process tied to cumulative clinical exposure and frequency of practice.^12^^,^^16^ However, it is important to acknowledge that different applications of POCUS may require differing levels of training and supervision, and reliance on “informal exposure” without a structured curriculum or deliberate practice may lead to uneven skill development across programs.^13^

The limited POCUS utilization and competency standards across IM programs are likely a complex and multifaceted issue. Thus, the lack of recommendations, coupled with POCUS findings, may limit its utilization. The Accreditation Council for Graduate Medical Education recognizes that IM residency programs may have different areas of focus and provides a broad recommendation for programs to develop competence in procedures required for practicing IM.^17^ However, it does not specify procedures or a specific number, and instead encourages programs to develop their own curricula for procedures. Thus, IM programs have varied procedural curricula and may limit the development of a uniform POCUS curriculum and its implementation in IM residency programs. This consistent trend likely reflects the frequent and mandatory use of obtaining venous (eg, difficult peripheral or central venous) or arterial (eg, arterial lines) access for patient safety or in volume assessment in critical care settings, aiming to better optimize hemodynamic status.

Several studies have called for standardized, competency-based POCUS training models in IM to ensure equity and proficiency.^14^^,^^15^ Despite its growing recognition as a core diagnostic tool, POCUS remains underutilized in IM compared with specialties such as EM and Critical Care, where it is embedded in core training standards.^18^^,^^19^ In addition, the Alliance for Academic IM and Society of Hospital Medicine have endorsed the inclusion of POCUS training in residency curricula.^20^^,^^21^ Yet, implementation across programs remains patchy and largely unstandardized.

Our findings highlight the limited and variable incorporation of POCUS in residency programs. We hope that our findings support the incorporation of competency-based curricula, ideally integrated across all PGY levels, and that these curricula are accompanied by simulation-based training, bedside feedback, and longitudinal assessments.^22^ Faculty development is equally essential. The shortage of trained faculty supervisors was a recurring barrier in our study, echoing previous literature identifying a lack of experienced faculty as a major bottleneck to POCUS growth in IM programs.^2^^,^^22^^,^^23^ Moreover, our findings support the need for credentialing pathways and maintenance-of-competency programs similar to those in Critical Care and EM. POCUS integration should be considered as part of IM residency accreditation standards, and institutions should be encouraged to include POCUS metrics in trainee evaluations. Collaborative networks between programs may help share curricula, simulation resources, and faculty expertise to bridge structural gaps.

Our study has limitations. Although this study aimed to capture a snapshot of POCUS utilization in various IM residency programs nationwide, the response rate was low. This might have been due to residents’ busy schedules at specific times during the study period, resulting in insufficient time to complete the survey. Consequently, the collected responses may not accurately reflect the entire IM resident population. The absence of representation of Western US States, due to lack of response, further limits the generalizability of these findings. Nevertheless, this study gathered responses from residents across diverse residency programs. These challenges could be addressed by including a similar survey at the end of the American College of Physicians’ annual IM In-Training Examinations to increase response rates across all regions. We also did not specifically include thoracic ultrasound as a standalone skill, which may have underrepresented its use. As noted by Peng et al^24^ in their study assessing POCUS training among Family Medicine residents in Canada, the rigidity of survey responses may have hindered the capture of some barriers not addressed in the survey. This survey evaluated self-reported confidence, which should not be equated with competence. Self-reported confidence is also subject to the Dunning-Kruger effect, a cognitive bias in which, in this study, residents might overestimate their skill in using POCUS.

## Conclusion

This study provides a framework for major IM professional medical societies to implement a nationwide standardized POCUS curriculum and training for IM residency programs in the United States. Our findings underscore the need for ongoing efforts from IM program leaders to ensure the implementation of dedicated POCUS elective time, the provision of more portable ultrasound machines, structured, hands-on, faculty-led bedside training, and credentialing pathways for POCUS. Larger prospective studies are required to assess whether these targeted POCUS interventions have led to increased confidence and competence among IM residents.

## CRediT authorship contribution statement

**Ugochukwu Ebubechukwu:** Writing – review & editing, Writing – original draft, Visualization, Supervision, Resources, Project administration, Methodology, Investigation, Formal analysis, Data curation, Conceptualization. **Onyinye Ugoala:** Writing – original draft, Visualization, Methodology, Data curation. **Chinenye Okafor:** Writing – original draft, Validation, Methodology, Formal analysis, Data curation. **Festus Ibe:** Writing – original draft, Visualization, Methodology, Data curation. **Duaa Mohamed:** Writing – original draft, Data curation. **Anderson Anuforo:** Writing – review & editing, Data curation. **Sahil Chaddha:** Writing – review & editing, Project administration, Data curation. **Ayesha Samad:** Writing – review & editing, Data curation. **Oboseh John Ogedegbe:** Writing – review & editing, Data curation. **Ahmad Abdelkhalek:** Writing – review & editing, Data curation. **Samy I. McFarlane:** Writing – review & editing, Supervision, Resources, Project administration. **Pooja Belligund:** Writing – review & editing, Writing – original draft, Supervision, Resources, Project administration, Investigation, Conceptualization.

## Declaration of competing interest

The authors declare no conflicts of interest.

## Appendix Supplementary materials

Image, application 1
