# Supplementary material for: Utilization of Point-of-Care Ultrasound in Routine Patient Care Among Internal Medicine Resident Physicians in the United States
Source: Am J Med Open. 2026 Apr 30;16:100133. doi: 10.1016/j.ajmo.2026.100133 (PMC13315898; doi:10.1016/j.ajmo.2026.100133)
Supplement: Supplementary file 1 [file mmc1.pdf]

<u>POINT-OF-CARE-ULTRASOUND (POCUS) QUESTIONNAIRE</u>

Hi, I am conducting a cross-sectional, multi-institutional study titled ***"Utilization of Point-of-Care Ultrasound in Routine Patient Care Among Internal Medicine Resident Physicians in the United States"*** across the nation. The purpose of this research is to assess respondents' knowledge of point-of-care ultrasound (POCUS), and to evaluate the practices and barriers to its effective utilization in their respective institutions.

You will be required to answer a few questions via this Google Questionnaire. The responses will be used for quality improvement and/or research purposes to enhance POCUS education in residency programs. ***It will take approximately 4-5 minutes to complete this questionnaire.***

There are no risks or compensations involved in participating in this study. All responses will be kept confidential. Any research or quality improvement data that is presented or published will be de-identified and anonymous.

Thank you for your participation.

1. Age (in years)
   a. 18-24
   b. 25-34
   c. 35-44
   d. ≥45

2. Sex
   a. Male
   b. Female

3. Specialty
   a. Internal Medicine
   b. Other (specify): ___________________

4. Level in training
   a. PGY-1
   b. PGY-2
   c. PGY-3

   d. PGY-4
   e. PGY-5

5. What type of residency program do you currently train in?
   a. University program
   b. University-affiliated program
   c. Community program

6. In which state is your residency program? (Drop down option to select state)

7. On average, how many patients do you care for in a week?
   a. <10
   b. 11-19
   c. 20-29
   d. 30-39
   e. 40-49
   f. ≥50

8. What prior training do you have with Point of care ultrasound or POCUS?
   a. Medical school
   b. Residency training
   c. Conferences:
   d. Simulation labs
   e. Online courses:
   f. Website
   g. Self-taught
   h. Other: ___________________

9. How many years of experience do you have with POCUS?
   a. 0-1
   b. 2-3
   c. 4-5
   d. >5

10. Do you have an ultrasound machine in every medical floor that you can use for POCUS?
    a. Yes
    b. No
    c. Not sure

11. Do you have an ultrasound medical director in your residency program that can provide leadership and education to help you acquire and or maintain POCUS skills?
    a. Yes
    b. No
    c. Not sure

12. Do you have a POCUS curriculum within your residency program that: (select all that apply)?

a. Can educate and train residents/fellows to use POCUS
b. Provides a credentialing pathway for untrained POCUS users
c. Provides a credentialing pathway for experienced POCUS users
d. Has a CME pathway to maintain credentialing?
e. Has a quality assurance program to review scans, problem solve and provide ongoing training

13. Approximately how many patients have you scanned in the last month:
    a. 0
    b. 1-2
    c. 3-4
    d. 4-5
    e. 5-6
    f. >7

14. Approximately how many patients have you scanned in the last 3 months:
    a. 0
    b. 1-3
    c. 4-7
    d. 8-10
    e. >10

15. Approximately how many patients have you scanned in the last 6 months:
    a. 0
    b. 1-4
    c. 5-8
    d. 9-12
    a. >12

16. Approximately how many patients have you scanned in the last 12 months:
    a. 0
    b. 1-5
    c. 6-10
    d. 11-15
    e. 16-20
    f. >20

17. What scans do you typically perform?
    a. E-FAST
    b. Focused cardiac
    c. IVC volume assessment
    d. Lung
    e. Soft Tissue
    f. Procedural
    g. Other (specify): ________________________

18. What scan do you feel less comfortable performing?

a. E-FAST
b. Focused cardiac
c. IVC volume assessment
d. Lung
e. Soft Tissue
f. Procedural
g. Other (specify): ________________________

19. What scans would you like to learn and perform?
    a. E-FAST
    b. Focused cardiac
    c. IVC volume assessment
    d. Lung
    e. Soft Tissue
    f. Procedural
    g. Other (specify): ________________________

20. What are the barriers that keep you from performing POCUS? (select all that apply)
    a. Lack of a machine
    b. Lack of an ultrasound medical director
    c. Lack of time
    d. Lack of confidence in your ability to obtain images
    e. Lack of confidence in your ability to interpret images
    f. Lack of a quality assurance program to verify image acquisition and interpretation
    g. Lack of experienced faculty (internal or external) for hands-on training and maintenance education
    h. Other: ____________________________________

21. What could be done to help you to utilize POCUS effectively? (select all that apply)
    a. Availability of an ultrasound machine in every ward
    b. Availability of a dedicated elective time for POCUS
    c. Incorporation of POCUS into medical education/training during the intern year of residency
    d. Availability of an ultrasound medical director
    e. Availability of an experienced faculty (internal or external) for hands-on training and maintenance education
    f. Availability of a quality assurance program to verify image acquisition and interpretation
    g. Other: ____

For each of the following statements, please circle the number that best corresponds to your response:

	Strongly Disagree	Disagree	Neutral	Agree	Strongly Agree
20. I am confident adjusting the basic knobs such as gain and depth	1	2	3	4	5
21. I am confident at selecting the correct probe for a given patient and purpose	1	2	3	4	5
22. I am confident in finding and obtaining vascular access with POCUS	1	2	3	4	5
23. I am confident at performing either a FAST exam and/or an EFAST exam	1	2	3	4	5
24. I am confident that I can diagnose pericardial effusion with POCUS	1	2	3	4	5
25. I am confident that I can diagnose tamponade with POCUS	1	2	3	4	5
26. I am confident that I can diagnose a pneumothorax with POCUS	1	2	3	4	5
27. I am confident that I can diagnose fluid in the peritoneum such as hemoperitoneum or ascites with POCUS	1	2	3	4	5
28. I am confident in my ability to assess left ventricular function with POCUS	1	2	3	4	5
29. I am confident in my ability to evaluate right ventricle function and volume overload with POCUS	1	2	3	4	5

30. I am confident in my ability to evaluate volume responsiveness with POCUS	1	2	3	4	5
31. I am confident in my ability to diagnose shock with POCUS	1	2	3	4	5

32. How confident are you in obtaining the following basic cardiac views using POCUS?

	Strongly Disagree	Disagree	Neutral	Agree	Strongly Agree
1. Parasternal Long Axis (PSLA) View	1	2	3	4	5
2. Parasternal Short Axis (PSSA) View					
-Mitral Valve Level	1	2	3	4	5
-Aortic Valve Level	1	2	3	4	5
3. Apical Views					
- Apical 4 Chamber (A4C) View	1	2	3	4	5
- Apical 5 Chamber (A5C) View	1	2	3	4	5
4. Subxiphoid (Subcostal) View	1	2	3	4	5
5. Inferior Vena Cava (IVC) View	1	2	3	4	5

33. I am confident in my ability to acquire images and interpret them with POCUS “putting it all together”	1	2	3	4	5

The original survey instrument (i.e., the POCUS questionnaire) was adapted from the Chan et al (https://jahse.med.utah.edu/wp-content/uploads/2021/06/Chan_Supplementary_Material_1.pdf). However, Chan et al. used the survey instrument in a 2-day POCUS course for fellows and practicing clinicians at a free-standing, university-affiliated children's hospital.

Given our desired population of interest (IM residents) and the need for a national survey, the survey was modified to be suitable for IM residents following discussions with ultrasound faculty members (an IM POCUS director (Pulmonary and Critical care attending) with many years of teaching POCUS experience, and additional IM POCUS Faculty and Chief Resident). The survey was also sent for an additional independent external review and modified as follows:

1. Age (in years), sex, specialty, level in training, and type of residency program were added to our survey instrument to obtain a baseline demographic of respondents.
2. "Where do you practice" in Chan et al.'s original survey was modified into "In which state is your residency program?" to capture the states and ensure multiple states were represented.
3. "How many years of experience do you have with POCUS?" was slightly modified to include discrete for streamlined data collection and analysis.
4. "Do you have a POCUS curriculum within your department that: (select all that apply)?" was modified into "Do you have a POCUS curriculum within your residency program that: (select all that apply)?" Additionally, options b, c, and d from Chan et al.'s survey were removed from our survey to focus on our target population (i.e., IM residents).
5. Additional options were included in questions 13, 14, and 16 in our survey to avoid overlapping responses.
6. Questions 18 ("What scan do you feel less comfortable performing?") and 19 ("What scans would you like to learn and perform?") were added/improved upon to better capture/assess the confidence levels with the different types of POCUS scans.
7. Options c, d, i, k, and l in question 16 of Chan et al.'s survey were also removed from our survey to focus on the identification of barriers to the utilization of POCUS on our target population (i.e., IM residents).
8. Finally, question 32 in our survey was included to capture the confidence levels of residents in obtaining basic cardiac views using POCUS.
